# Supplementary material for: Role of Non-Binding T63 Alteration in IL-18 Binding
Source: Int J Mol Sci. 2024 Dec 3;25(23):12992. doi: 10.3390/ijms252312992 (PMC11641284; doi:10.3390/ijms252312992)
Supplement: Supplementary file 1 [file ijms-25-12992-s001.zip › TableS1-Protein sequence alignment analysis.pdf]

Table S1. Protein sequence alignment analysis. Full-length mammalian IL-18 proteins were aligned with default setting of blastp and ClustalO by UniProt.

**Data**	**Mammalian IL-18**				
	**Human**	**Rat**	**Bovine**	**Horse**	**Mouse**
**Uniprot ID**	Q14116	P97636	Q9TU73	Q9XSQ7	P70380
**Amino acid length**	193	194	193	193	192
**Identical position***	193	121	150	153	122
**E-value**	5.7e-138	1.6e-80	1.2e-107	2.2e-110	3e-80
**Score**	1004	627	805	823	625
**%Identity***	100.0	62.1	77.7	79.3	62.3
**Similar position***	0	40	30	27	40

* Compared to human IL-18
