# Supplementary material for: Role of Non-Binding T63 Alteration in IL-18 Binding
Source: Int J Mol Sci. 2024 Dec 3;25(23):12992. doi: 10.3390/ijms252312992 (PMC11641284; doi:10.3390/ijms252312992)
Supplement: Supplementary file 1 [file ijms-25-12992-s001.zip › TableS2-List of model.pdf]

Table S2. List of the modeled proteins. The initial structures were generated by Phyre2 webserver. RMSD values were compared between the backbone proteins at N, Cα, and C of the initial structure and template.

**Engineered IL-18**	**Coverage (%)**	**Identity (%)**	**RMSD (Å)**	**Template**
Wild type	100	100	0.000	1J0S
E6A	100	99	0.000	1J0S
E6K	100	99	0.000	1J0S
T63A	100	99	0.000	1J0S
M33Q	100	99	0.000	1J0S
E6K/T63A	100	99	0.000	1J0S
E6K/T63V	100	99	0.000	1J0S
E6K/T63I	100	99	0.000	1J0S
E6K/T63L	100	99	0.000	1J0S
E6K/T63M	100	99	0.000	1J0S
E6K/T63S	100	99	0.000	1J0S
E6K/T63N	100	99	0.000	1J0S
E6K/T63Q	100	99	0.000	1J0S
E6K/T63G	100	99	0.000	1J0S
E6K/T63C	100	99	0.000	1J0S
E6K/T63P	100	99	0.000	1J0S
E6K/T63F	100	99	0.000	1J0S
E6K/T63W	100	99	0.000	1J0S
E6K/T63Y	100	99	0.000	1J0S
E6K/T63R	100	99	0.000	1J0S
E6K/T63H	100	99	0.000	1J0S
E6K/T63K	100	99	0.000	1J0S
E6K/T63D	100	99	0.000	1J0S
E6K/T63E	100	99	0.000	1J0S
