# Supplementary figures and images for: Role of Non-Binding T63 Alteration in IL-18 Binding
Source: Int J Mol Sci. 2024 Dec 3;25(23):12992. doi: 10.3390/ijms252312992 (PMC11641284; doi:10.3390/ijms252312992)

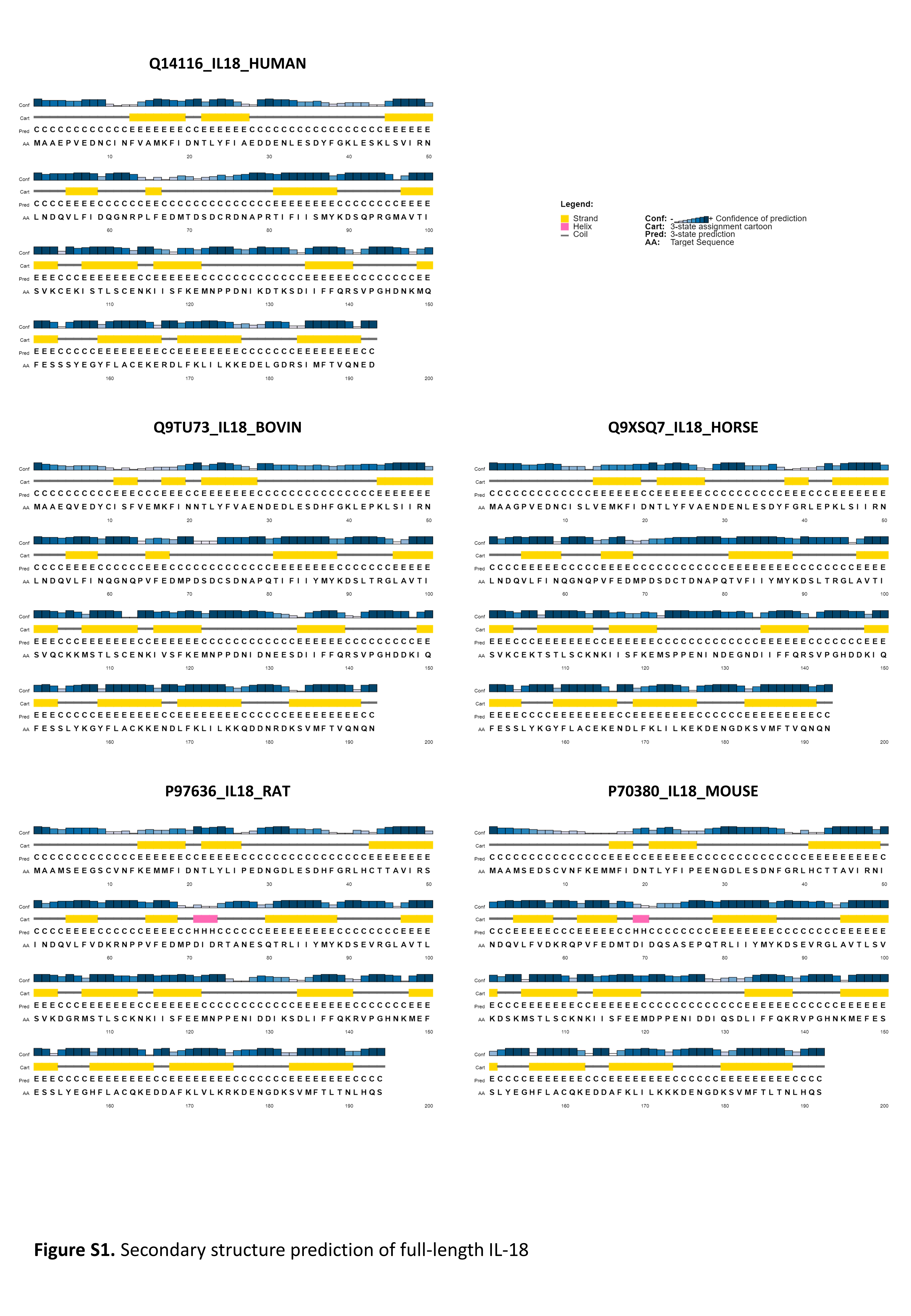

Supplement: Supplementary file 1 [file ijms-25-12992-s001.zip › Figures S1-S3/FigS1-Secondary structure.tif]

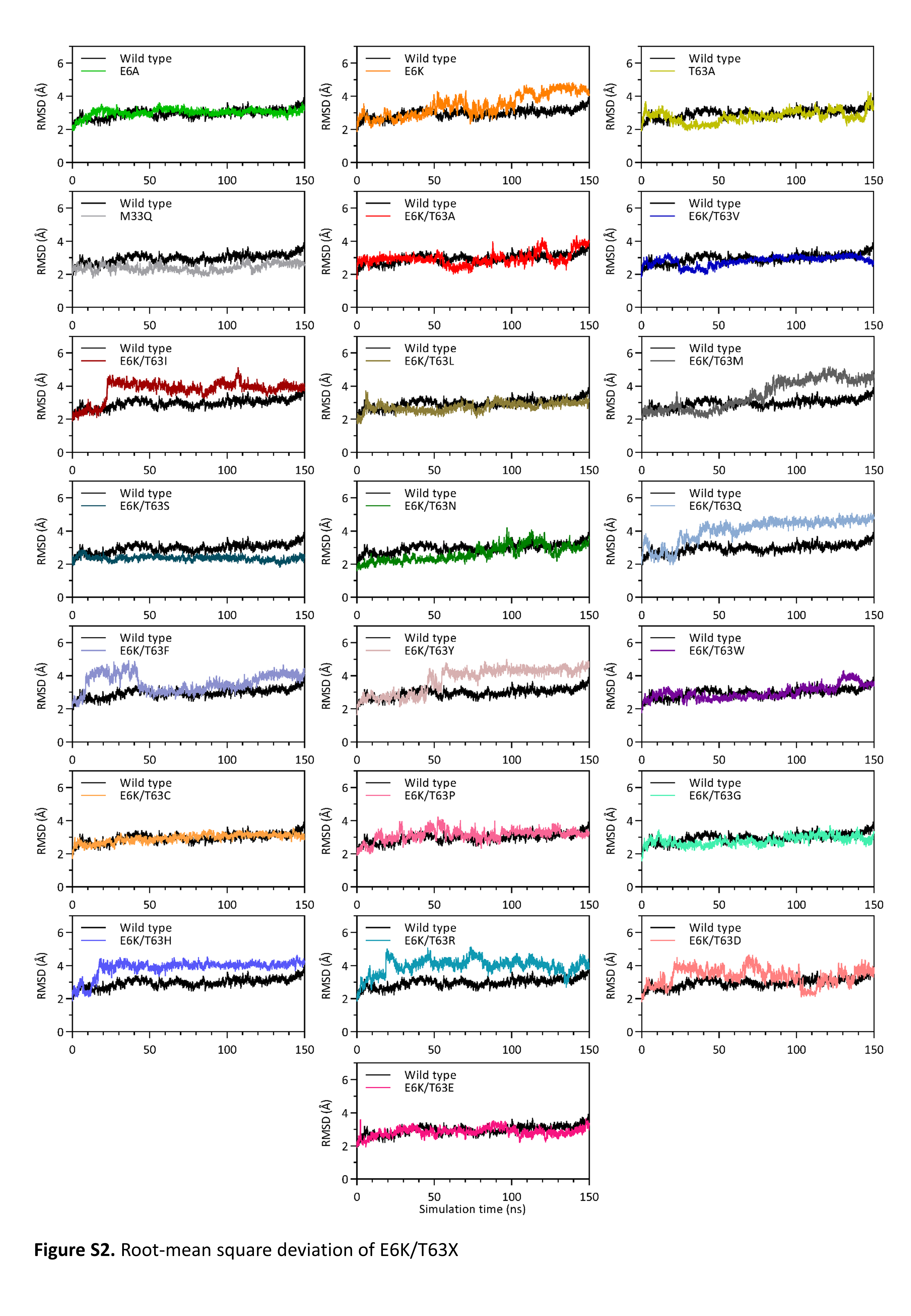

Supplement: Supplementary file 1 [file ijms-25-12992-s001.zip › Figures S1-S3/FigS2-RMSD.tif]

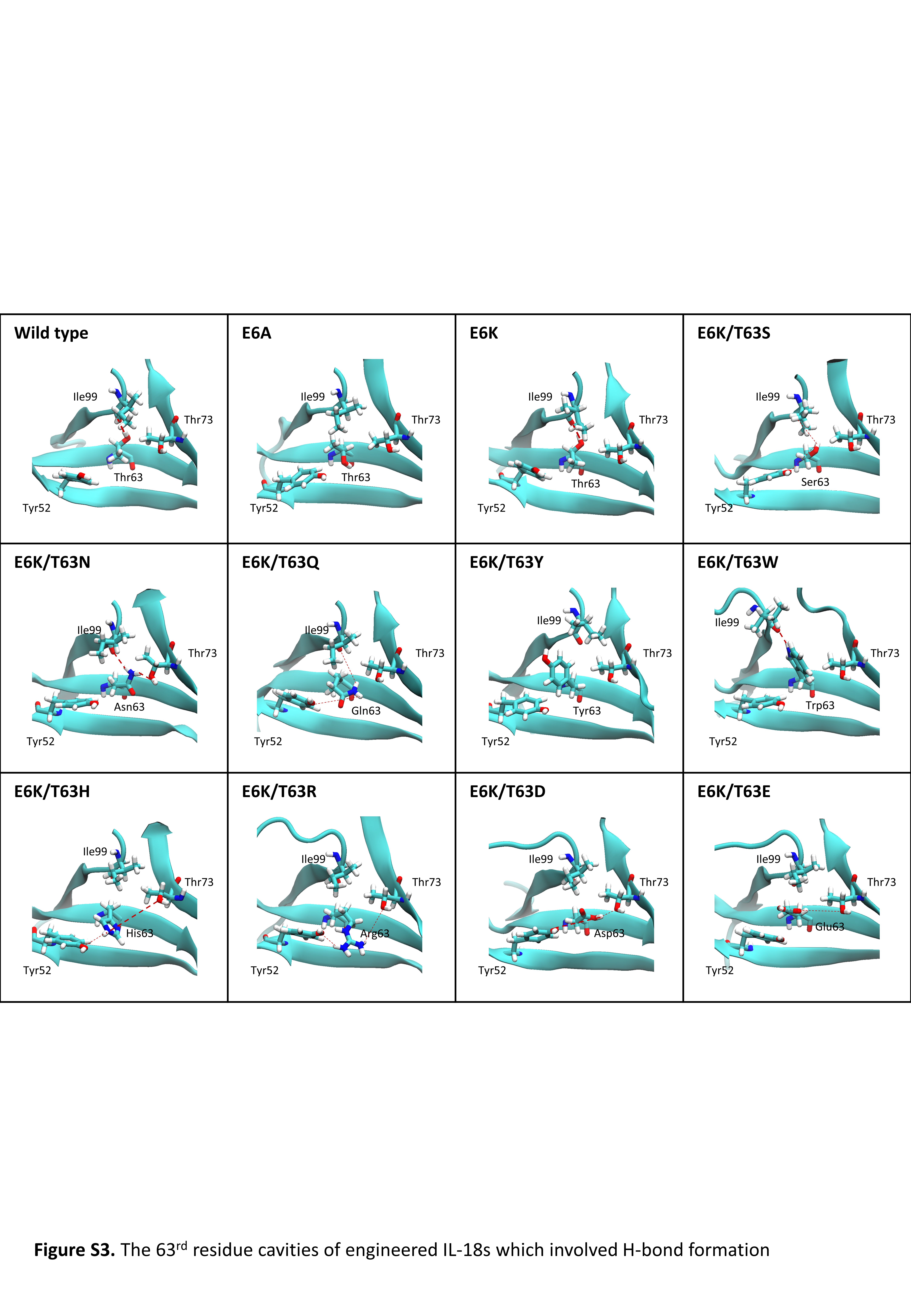

Supplement: Supplementary file 1 [file ijms-25-12992-s001.zip › Figures S1-S3/FigS3-The 63rd residue cavity.tif]
